# Supplementary material for: Immunomorphological Pattern of Molecular Chaperones in Normal and Pathological Thyroid Tissues and Circulating Exosomes: Potential Use in Clinics
Source: Int J Mol Sci. 2019 Sep 11;20(18):4496. doi: 10.3390/ijms20184496 (PMC6770414; doi:10.3390/ijms20184496)
Supplement: Supplementary file 1 [file ijms-20-04496-s001.pdf]

Supplementary Table S1. Clinical Characteristics of Patients					
**Disease**	**Sex**	**Age**	**Thyroid weight (g)**	**Nodular localization**	**Nodular size (diam. max; cm)**
BG	F	68	60		
BG	M	49	160		
BG	F	47	80		
BG	F	70	25		
BG	F	58	130		
BG	M	51	30		
BG	F	59	80		
BG	F	56	60		
BG	F	58	40		
BG	F	68	110		
BG	F	59	50		
BG	M	76	67		
BG	F	69	20		
BG	F	65	75		
BG	F	65	60		
BG	F	47	90		
BG	M	59	65		
BG	F	59	50		
PC	F	47	12	Left lobe	1,1
PC	F	57	100	Left lobe	0,75
PC	F	36	30	Left lobe	3
PC	F	50	20	Right lobe	1,2
PC	F	66	50	Left lobe	1
PC	F	42	120	Right lobe	2,1
PC	F	55	50	Left lobe	3
PC	F	43	20	Isthmus	2,8
PC	M	62	20	Left lobe	1
PC	F	61	15	Right lobe	3
PC	F	25	70	Right lobe	2
PC	F	56	30	Left lobe	1,8
PC	M	53	45	Left lobe	2,1

BG: Benign goiter; PC: Papillary Carcinoma; F: female; M: male;
